# Lipid profiling of the therapeutic effects of berberine in patients with nonalcoholic fatty liver disease

**DOI:** 10.1186/s12967-016-0982-x

**Published:** 2016-09-15

**Authors:** Xinxia Chang, Zhe Wang, Jinlan Zhang, Hongmei Yan, Hua Bian, Mingfeng Xia, Huandong Lin, Jiandong Jiang, Xin Gao

**Affiliations:** 1 Department of Endocrinology and Metabolism, Zhongshan Hospital, Fudan University, Shanghai, China; 2 Institute of Metabolic Disease of Fudan University, Shanghai, China; 3 Institute of Materia Medica, Chinese Academy of Medical Sciences, and Peking Union Medical College, Beijing, 100050 China

**Keywords:** Berberine, Nonalcoholic fatty liver disease, Lipidomic approach, Sphingolipids, Ceramide

## Abstract

**Background:**

We recently demonstrated a positive effect of berberine on nonalcoholic fatty liver disease patients after 16 weeks of treatment by comparing mere lifestyle intervention in type 2 diabetes patients with berberine treatment, which decreased the content of hepatic fat. However, the potential mechanisms of the clinical effects are unclear. We used a lipidomic approach to characterize the state of lipid metabolism as reflected in the circulation of subjects with nonalcoholic fatty liver disease (NAFLD) before and after berberine treatment.

**Methods:**

Liquid chromatography–mass spectrometry evaluated the various lipid metabolites in serum samples obtained from the participants (41 patients in the berberine group and 39 patients in the mere lifestyle intervention group) before and after treatment.

**Results:**

A total of 256 serum lipid molecular species were identified and quantified. Both treatments regulated various types of lipids in metabolic pathways, such as free fatty acids, phosphoglycerides and glycerides, in metabolic pathways, but berberine induced a substantially greater change in serum lipid species compared with mere lifestyle intervention after treatment. Berberine also caused obvious differences on ceramides. Berberine treatment markedly decreased serum levels of ceramide and ceramide-1-phosphate.

**Conclusions:**

Berberine altered circulating ceramides, which may underlie the improvement in fatty liver disease.

*ClinicalTrials.gov* NCT00633282, Registered March 3, 2008

**Electronic supplementary material:**

The online version of this article (doi:10.1186/s12967-016-0982-x) contains supplementary material, which is available to authorized users.

## Background

Berberine (BBR) is an alkaloid that was originally isolated from Huanglian (Coptischinensis). BBR is used as antimicrobial in China. Recent studies have demonstrated beneficial effects of BBR on serum lipids and glucose metabolism [1–3]. BBR exerted an anti-hyperlipidemia effect of lowering total cholesterol (TC), triglyceride (TG), low-density lipoprotein cholesterol (LDL-c) levels in patients [2]. The cholesterol-lowering mechanism of BBR was different from that of statins. Berberine elevated the LDLR expression by stabilizing LDLR message ribonucleic acid(mRNA) [2] and blocking proprotein convertase subtilisin/kexin type 9 (PCSK9) transcription [4, 5], but statins upregulated PSCK9 gene expression [6]. BBR is also an effective anti-diabetes agent.BBR significantly lowered fasting blood glucose (FBG), hemoglobin A1C, triglyceride, and insulin levels in patients with type 2 diabetes mellitus (T2DM) in the clinical study [7].

The liver plays a vital role in lipid metabolism and glucose homeostasis. Nonalcoholic fatty liver disease (NAFLD) is associated with insulin resistance and the development of type 2 diabetes [8]. The effect of BBR on regulating serum cholesterol and triglyceride suggested that BBR played a central role in decreasing hepatic fat content. Our previous study [9] demonstrated that BBR decreased hepatic fat content by 57.2 %, and reduced serum lipids and liver enzymes, which indirectly indicated that BBR improved liver inflammation. However, the mechanism of reducing hepatic fat content is not known.

Increased TG accumulation in the liver is the pathophysiological hallmark of NAFLD. Some studies suggest that the lipid metabolism perturbations in NAFLD are more complex. A comprehensive lipidomics study demonstrated that substantial changes in other lipid classes, such as cholesterol and specific phospholipids in the liver may play a role in the pathogenesis of NAFLD and the development of NASH [10]. NAFLD is also associated with several changes in circulating lipidomics, such as an increase in the ratio of monounsaturated fatty acids: vs saturated fatty acids and a significant decrease in circulating levels of the essential fatty acids linoleic acid (18:2 n6) and alpha-linolenic acid (18:3 n3) across multiple lipid classes. The lipogenic activity levels off or declines modestly with progression to nonalcoholic steatohepatitis (NASH), but lipoxygenase (LOX) activity increases [11]. Therefore, circulating lipidomics are closely associated with fat deposits in hepatocytes. Whether the compositions of the lipids are harmful and whether the changes of serum lipidomics are related with berberine treatment effect are not well identified. This study used a comprehensive lipidomic approach: (1) to quantify the absolute and relative amounts of free fattyacids (FFAs), glycerolipids (GL), glycerophospholipids (GP) and sphingolipids (SP) in subjects with NAFLD before and after berberine treatment and lifestyle intervention and (2) to compare the distribution of fatty acids within each of these classes in these groups of subjects.

## Methods

### Participants

The detailed design of this study was previously published [9]. Briefly, a randomized, parallel-controlled, open-label clinical trial was conducted in three medical centers for the treatment of NAFLD patients with impaired glucose regulation (IGR) or T2DM with lifestyle intervention (LSI) with or without BBR (NIH Registration number: NCT00633282). The trial design conformed to the revised CONSORT standards for the reporting of randomized trials. Eligible adults were identified and recruited from unsolicited referrals to the three participating clinical centers from March 2008 to August 2011. Hepatic fat content (HFC) was assessed using proton magnetic resonance spectroscopy (^1^H MRS). Subjects who met all enrollment criteria were randomly assigned to one of the two groups for the 16-week clinical trial, Group A- LSI or Group B-LSI plus BBR (0.5 g, t.i.d.). BBR (berberine^®^, Huashi Pharmaceuticals Shanghai, China, Inc.) was administered orally at a 0.5 g dose 30 min before meals, three times daily (according to the Chinese Pharmacopeia [12]. LSI (including dietary modification and exercise) was performed following standardized recommendations [13]. All participants were required to fast overnight (12 h) before participating in a physical examination by trained staff and physicians using standard protocols. Blood samples were drawn after an overnight fast and immediately centrifuged. The samples were frozen immediately and stored at −80 °C until assayed. These samples were used for the final lipidomics analysis. Table 1 summarizes the detailed characteristics of these 80 patients at baseline and at the end of follow-up. The following main reasons determined the exclusion of the original participants from the present analysis: (1) incomplete information and (2) insufficient blood samples. The ethics committee of Zhongshan Hospital, Fudan University approved the study, which was conducted in accordance with the guidelines of the Declaration of Helsinki. Written informed consent was obtained from each patient.

**Table 1:** Changes of clinical and biochemical parameters after treatment

	BBR plus LSI (n = 41)		*P* value	LSI (n = 39)		*P* value
	Baseline	After 16 w		Baseline	After 16 w	
Sex (M/F)	26/15			20/19		
Age (years)	51.2 ± 9.4			50.8 ± 10.4		
Weight (kg)	77.0 ± 15.4	72.8 ± 13.3	<0.01	75.9 ± 10.6	74.0 ± 11.1	<0.05
BMI (kg/m^2^)	27.4 ± 4.1	25.5 ± 3.3	<0.01	27.3 ± 3.0	26.0 ± 5.9	<0.05
Waist (cm)	94.5 ± 11.1	89.7 ± 9.7	<0.01	93.1 ± 7.3	90.8 ± 8.5	<0.05
HFC (%)	30.3 (22.2–44.0)	13.6 (9.3–17.4)	<0.01	28.7 (21.9–46.8)	20.3 (11.3–33.0)	<0.05
Blood glucose (mmol/L)						
0 min	6.4 ± 0.9	6.1 ± 1.3	<0.01	6.2 ± 1.0	6.2 ± 0.9	0.47
30 min	11.1 ± 1.7	9.9 ± 1.9	<0.05	11.0 ± 1.7	10.4 ± 2.1	0.13
60 min	13.1 ± 2.5	11.1 ± 2.6	<0.05	13.0 ± 2.6	11.9 ± 3.1	<0.05
120 min	11.2 ± 3.0	8.6 ± 2.8	<0.05	10.8 ± 3.3	10.1 ± 2.9	0.14
180 min	7.2 ± 2.3	5.4 ± 1.6	<0.05	6.5 ± 2.8	5.9 ± 2.3	<0.05
AUCg	42.4 ± 7.6	36.3 ± 8.4	<0.05	41.2 ± 8.4	39.1 ± 9.3	<0.05
HbA1c (%)	6.4 ± 0.7	6.0 ± 0.4	<0.01	6.3 ± 0.7	6.2 ± 0.7	0.11
Serum insulin (mU/mL)						
0 min	13.4 (8.5–16.2)	10.8 (8.5–14.1)	0.97	3.4 (8.6–18.6)	12.4 (7.1–19.1)	0.89
30 min	41.0 (30.6–62.2)	47.9 (33.3–88.5)	0.34	59.9 (33.2–83.4)	54.2 (28.8–77.7)	0.28
120 min	69.9 (47.5–100.9)	68.0 (41.2–103.1)	0.75	78.1 (57.1–132.7)	74.6 (45.7–117.9)	0.23
HOMA-IR	3.6 (2.4–4.1)	2.8 (2.2–3.9)	0.21	3.7 (2.5–5.0)	3.4 (1.8–5.1)	0.32
HOMAβ	88.2 (54.3–114.4)	93.1 (64.1–129.6)	0.12	97.7 (62.2–151.1)	100.0 (56.8–152.6)	0.69
ΔI30/ΔG30	8.0 (4.1–13.6)	10.7 (5.3–20.3)	*P* < 0.05	9.4 (5.0–16.6)	9.3 (3.6–18.6)	0.65
TC (mmol/L)	5.3 ± 0.9	4.6 ± 1.0	*P* < 0.01	5.0 ± 0.8	5.0 ± 0.8	0.92
TG (mmol/L)	2.2 ± 1.2	1.6 ± 0.9	*P* < 0.05	2.0 ± 0.8	1.9 ± 1.1	0.69
HDL-c (mmol/L)	1.2 ± 0.2	1.2 ± 0.3	0.65	1.2 ± 0.3	1.2 ± 0.2	0.55
LDL-c (mmol/L)	3.2 ± 0.9	3.0 ± 0.9	*P* < 0.05	3.0 ± 0.8	3.0 ± 0.8	0.73
APO-A(g/L)	1.3 (1.1–1.4)	1.2 (1.0–1.3)	*P* < 0.05	1.3 (1.0–1.4)	1.3 (1.1–1.5)	0.89
APO-B(g/L)	1.0 ± 0.2	0.9 ± 0.2	*P* < 0.05	1.0 ± 0.2	1.0 ± 0.2	0.28
APO-E (mg/L)	48.0 (37.0–57.3)	42.5 (36.0–49.7)	0.08	44.0 (37.9–52.3)	44.0 (38.5–56.5)	0.94
LP(a) (mg/L)	104.0 (50.0–248.0)	101.0 (49.5–272.0)	*P* < 0.05	155.0 (78.0–218.0)	160.0 (90.3–263.0)	0.23
Liver enzyme (U/L)						
ALT	36.0 (24.5–47.0)	21.0 (13.5–29.5)	*P* < 0.01	34.0 (25.3–46.5)	21.0 (15.0–36.3)	*P* < 0.01
AST	25.0 (20.0–32.0)	19.0 (16.0–22.5)	*P* < 0.01	25.0 (20.0–30.0)	20.5 (15.8–27.0)	*P* < 0.05
γ-GT	39.0 (26.0–67.5)	30.0 (23.0–43.0)	*P* < 0.01	33.0 (22.0–56.5)	27.5 (20.5–48.8)	*P* < 0.05

The data were presented as the mean ± SD, except for skewed variables, which were presented as the median with the interquartile range given in parentheses

*LSI* lifestyle intervention, *BBR plus* *LSI* berberine treatment plus lifestyle intervention, *BMI* body-mass index, *HFC* hepatic fat content, *TC* total cholesterol, *TG* triglyceride, *HDL-c* high-density lipoprotein cholesterol, *LDL-c* low-density lipoprotein cholesterol, *ALT* alanine aminotransferase, *AST* aspartate aminotransferase, *γ-GT* γ-glutamyltransferase

### Materials

All lipid standards were purchased from Avanti Polar Lipids (Alabaster, AL, USA). Organic residue grade methanol, MS grade acetonitrile and HPLC grade methyl-tert-butyl ether (MTBE) were purchased from Mallinckrodt BakerInc. (Phillipsburg, NJ, USA). HPLC grade isopropyl alcohol and chloroform were purchased from Honeywell Inc. (Muskegon, MI, USA). Formic acid of analytical grade was obtained from TEDIA Company, Inc. (Fairfield, OH, USA). Ammonium formate and ammonium acetate (purity 99.99 %) were purchased from Sigma-Aldrich (St. Louis, MO, USA). Ultra-pure water was prepared using a Milli-Q purification system (Millipore, Bedford, MA, USA).

### Method of lipid profiling

Free fatty acids were determined according to our previous study [14]. Samples were first extracted using reverse phase SPE and analyzed in an Agilent 6410B Triple Quadrupole LC–MS after pre-column derivatization.

Sphingolipids, phosphoglycerides and glycerides were determined in our lipid profiling platform [15] with a slight modification Plasma (0.1 mL) was transferred into a glass tube containing 20 μL of sphingolipid internal standards. Methanol (1.5 mL) was added to the tube, and the sample was vortexed for 10 s. Methyl-tert-butyl ether (MTBE) (5 mL) was added, and the sample was vortexed again for 15 min. Phase separation was induced by the addition of 1.5 mL of MS-grade water. Samples were incubated for 10 min at room temperature, and the tube was centrifuged at 4500 r/min for 10 min. The organic supernatant was collected, and the lower phase was re-extracted with 2 mL of the solvent mixture (MTBE:methanol:water, 10:3:2.5). The pooled organic supernatant was collected and dried under a gentle nitrogen stream. The dried extracts were re-dissolved in 100 μL of methanol/chloroform (1:1, v/v) containing internal standards of phosphoglycerides, glycerides and sphingomyelins for analysis. A 50-μL aliquot was used for Agilent 6410B Triple Quadrupole LC–MS testing to analyzes phingolipids, and another 50 μL were used for Thermo Scientific HPLC-LTQ/FTICRMS testing to analyze phosphoglycerides, glycerides and sphingomyelins.

Chromatographic separation was performed for sphingolipids testing using a SpectraC8SR column (150 × 3.0 mm; 3 μm particle size; Peeke Scientific, Redwood City, CA, USA). The column temperature was 40 °C. Mobile phase A was comprised of 1 mM ammonium formate in water containing 0.1 % formic acid. Mobile phase B was comprised of 1 mM ammonium formate in methanol containing 0.1 % formic acid. The gradient was programmed as follows: 0–10 min, 80–100 % B; 10–18 min, 100 % B; 18–18.1 min, 100–80 % B; and 18.1–25 min, 80 % B. The flow rate was 0.5 mL/min. The injection volume was 3 μL. The parameters for electrospray ionization tandem MS in positive ion mode were as follows: gas temperature, 350 °C; gas flow rate, 10 L/min; nebulizer, 30 psi; and capillary voltage, 4000 V. Multiple reaction monitoring was performed using the characteristic precursor-to-production transitions, optimized fragmentor voltages, and collision energies.

The surveyor HPLC system was equipped with an X terra MS C8 column (100 × 2.1 mm; 3.5-μm particle size; Waters, Milford, MA, USA) for phosphoglycerides, glycerides and sphingomyelins testing. Mobile phase A was comprised of 0.1 % formic acid in water containing 2 mM ammonium acetate. Mobile phase B was comprised of 2-propanol/acetonitrile (2:5, v/v) containing 2 mM ammonium acetate and 0.1 % formic acid. The flow rate was 0.35 mL/min. The gradient was programmed consecutively as follows: 0–1 min, 10 % B; 1–2 min, 10–30 % B2-4 min, 30–50 % B; 4–8 min, 50–70 % B; 8–12 min, 70–100 % B; and 12–24 min, 100 % B. The oven temperature was 40 °C. The injection volume was 10 μL. The LTQ-FT was run in full-scan mode at 100,000 resolution ranging from *m/z* 50–1200 with the following MS parameters: sheath gas flow rate, 50 arb; aux gas flow rate, 20 arb; sweep gas flow rate, 3 arb; and capillary temperature, 275 °C in positive and negative mode. The positive mode used a spray voltage of 4.5 kV, capillary voltage of 35.0 V, and tube lens of 120 V. The negative mode used a spray voltage of −4.0 kV, capillary voltage of −35.0 V, and tube lens of −120 V.

Sphingolipids were identified based on retention time using authentic standards and quantified using standard curve samples. The identification and quantitation of other lipids was performed using the lipid data analyzer (LDA) software package (Graz University of Technology, Graz, Austria).

### Statistical analysis

Variables are expressed as the means ± SD or medians (quartile). Differences between groups were analyzed using Student’s *t* test (for data that were normally distributed) or the Mann–Whitney test (for data that were not normally distributed) using SPSS 18.0 software (SPSS Inc., Chicago, IL, USA). A *P* value less than 0.05 was considered significant. Orthogonal partial least squares discriminant analysis (OPLS-DA) was used to visually discriminate between groups. Lipid profiling data were mean-centered and Pareto-scaled using Simca P + 12.0.1 (Umetrics, Umeå, Sweden) to reduce noise and artifacts. The quality and predictability of each OPLS-DA model was evaluated using R2Y (cum) and Q2 (cum) values, respectively. The following criteria for each potential biomarker were used: (1) A variable importance in projection greater than one; (2) The jack-knife uncertainty bar excluded zero; and (3) The absolute value of *P* corr in the S-plot was greater than 0.58 [16].

## Results

### The general characteristics of the subjects at baseline

Serum samples, which were subjected to the lipidomics analyses, were obtained from 41 BBR-treated patients and 39 LSI patients at baseline and at the end of treatment. Table 1 summarizes the detailed characteristics of the 80 patients at baseline. There were no significant differences in the clinical characteristics between groups at baseline, which suggests that the two groups were well matched in demographic profiles, HFC and other baseline characteristics (Table 1).

### Berberine significantly influenced hepatic fat content and energy metabolism

After 16 w treatment, HFC decreased by 55.1 % in the BBR group (*P* = 0.00) and by 29.3 % in the LSI group (*P* < 0.05; Table 1). BBR caused a greater reduction in HFC as compared to that with LSI alone (*P* = 0.021; Additional file 1: Table S1). Liver enzymes, such as ALT, AST and γ-GT were not significantly different between BBR and LSI groups at the 16th week (Additional file 1: Table S1).

Body weight, waist, body mass index (BMI), HFC, blood glucose, HbA1c, ΔI30/ΔG30, serum cholesterol, triglyceride, LDL-c, apoA/B, LP(a) and liver enzymes were significantly decreased after 16 weeks of BBR treatment (*P* < 0.05) (Table 1). BBR exhibited greater decreases in body weight, BMI, waist, HFC, serum cholesterol and triglycerides compared with LSI alone (Additional file 1: Table S1), which demonstrates a clearly significant benefit of BBR on metabolism. Additional file 2: Figure S1 shows the line graph of the glucose tolerance test (0–3 h). BBR reduced the area under the OGTT curve [−5.9(−6.9 to −4.8) vs. −4.0(−4.6 to −1.9), *P* = 0.041] more than lifestyle intervention alone.

### Lipid profiling of berberine treatment and lifestyle intervention alone

**Fig. 1:**
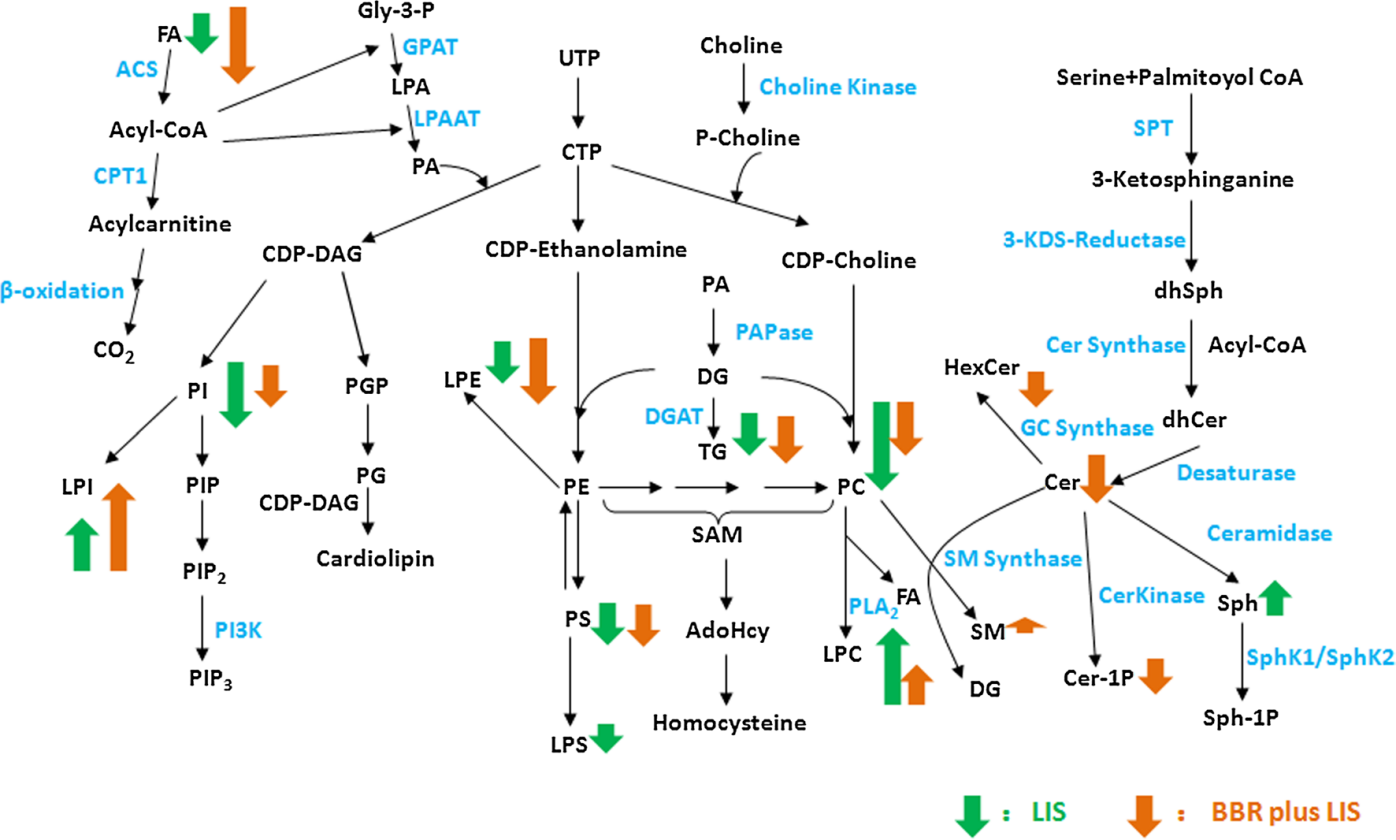
Lipid metabolic pathways and the regulation of berberine on the patients with IGR/Type 2 diabetes and nonalcoholic fatty liver disease

Sixty-one free fatty acids, 54 sphingolipids, 86 phosphoglycerides and 55 glycerides were successfully identified and quantified. Subsequent comprehensive statistical analyses identified lipid variations between the groups. Table 2 shows lipids with significant differences between groups after Student’s *t* test and Mann–Whitney tests. Figure 1 shows the effect of berberine and lifestyle intervention on lipid metabolic pathways. Berberine altered lipid metabolism, and this effect was related to a variety of lipid types.

**Table 2:** Lipid items significantly changed in response to berberine treatment and lifestyle intervention

	BBR plus LSI (n = 41)		*P* value	LSI (n = 39)		*P* value
	Baseline	16 w		Baseline	16 w	
Free fat acid						
FA(15:1)	3.5 ± 2.3	2.5 ± 1.5	0.029	3.1 ± 2.3	2.9 ± 3.0	0.689
FA(16:1)	2.5 (1.5–3.5)	1.5 (1.1–2.4)	0.006	2.1 (1.3–3.5)	1.2 (0.9–2.4)	0.013
FA(16:2)	1.6 (0.7–2.2)	0.7 (0.5–1.2)	0.008	1.1 (0.7–2.5)	0.6 (0.5–1.3)	0.028
FA(16:3)	1.3 ± 1.0	0.9 ± 0.6	0.026	1.3 ± 1.2	1.1 ± 1.1	0.453
FA(18:1)	2.7 (1.7–4.1)	2.0 (1.5–3.3)	0.043	2.4 (1.7–3.4)	1.6 (1.1–2.9)	0.054
FA(18:2)	2.3 (1.3–3.6)	1.2 (0.8–1.8)	0.005	1.6 (1.1–3.0)	1.4 (0.8–1.7)	0.010
FA(20:2)	2.4 (1.3–4.1)	1.7 (1.0–2.8)	0.024	1.9 (1.3–2.9)	1.4 (1.0–2.1)	0.028
FA(20:3)	2.1 (1.3–4.3)	1.4 (0.9–2.1)	0.003	1.7 (1.3–3.3)	1.3 (1.0–1.9)	0.031
FA(20:4)	1.6 (0.8–3.0)	0.8 (0.6–1.5)	0.005	1.2 (0.8–2.2)	0.9 (0.6–1.5)	0.081
FA(20:5)	1.3 (0.6–3.3)	0.7 (0.4–1.21)	0.006	1.3 (0.7–3.3)	0.9 (0.5–2.2)	0.176
FA(22:4)	2.8 ± 1.9	2.0 ± 1.6	0.038	2.7 ± 2.4	2.2 ± 1.8	0.294
FA(22:5)	1.5 (0.9–2.4)	1.1 (0.7–1.7)	0.032	1.4 (1.0–2.0)	1.2 (0.9–1.8)	0.230
FA(22:6)	1.3 (0.9–2.9)	1.0 (0.6–1.7)	0.017	1.3 (0.9–2.8)	1.1 (0.7–2.0)	0.101
FA27	0.9 (0.6–1.6)	0.8 (0.5–1.3)	0.381	1.0 (0.7–1.6)	0.7 (0.5–1.1)	0.040
FA3	1.0 (0.7–1.4)	0.6 (0.4–1.2)	0.018	1.1 (0.6–1.7)	0.8 (0.6–1.2)	0.057
FA40	2.1 ± 1.6	1.4 ± 1.0	0.011	2.2 ± 1.9	1.8 ± 1.5	0.303
FA48	2.2 (1.3–4.4)	1.4 (0.9–2.1)	0.020	1.8 (1.2–4.2)	1.2 (0.9–2.8)	0.072
FA51	2.9 (2.01–4.8)	1.9 (1.3–3.0)	0.004	2.5 (1.6–3.6)	1.9 (1.4–2.6)	0.029
FA(α-18:3)	1.9 (1.1–3.6)	1.0 (0.8–1.4)	0.003	1.8 (1.2–2.6)	1.0 (0.7–1.5)	0.002
FA(γ-18:3)	1.7 (1.0–2.8)	0.9 (0.7–1.2)	0.001	1.3 (0.8–2.3)	0.9 (0.7–1.7)	0.842
Sphingolipid (pmol/ml)						
Sph(d18:1)	15.3 (6.1–38.1)	11.3 (4.7–22.8)	0.139	20.4 (7.1–35.5)	10.7 (4.8–19.0)	0.050
Cer(d18:1/18:0)	41.1 (30.4–52.0)	31.8 (25.1–43.6)	0.033	37.7 (33.3–50.6)	34.8 (27.1–47.5)	0.576
Cer(d18:1/18:0)-1-p	472.4 (337.2–668.4)	423.6 (327.1–497.6)	0.033	491.5 (360.7–635.4)	459.8 (328.5–542.1)	0.734
Cer(d18:1/20:0)	103.7 (67.0–121.5)	87.1 (67.3–103.1)	0.047	87.1 (80.9–109.3)	85.5 (72.9–104.0)	0.398
Cer(d18:1/28:0)-1-P	88.3 (68.4–114.5)	81.4 (60.0–107.4)	0.219	91.1 (73.2–119.9)	95.9 (78.3–128.5)^#^	0.215
SM(d18:1/12:0)	1102.8 (561.7–1328.7)	1131.0 (615.0–1342.1)	0.066	1495.6 (921.5–1717.5)*	1794.1 (1221.6–2473.0)	0.854
SM(d18:1/16:1)	266084.4 (183,325.4–335932.6)	213,504.2 (151,956.4–266,787.1)	0.019	247,807.9 (168,113.3–334,244.5)	225,052.0 (160,531.6–291,093.4)	0.247
SM(d18:1/24:4)	1810.8 (1290.7–2388.9)	2426.6 (1756.9–3651.7)	0.040	1389.6 (916.5–2164.5)	1782.6 (1215.9–2228.4)	0.131
Phosphoglyceride (pmol/ml)						
LPC(14:0)	766.1 ± 533.2	508.5 ± 278.9	0.008	777.1 ± 562.5	651.7 ± 344.9^#^	0.239
LPC(16:1)	2149.7 (1322.3–2461.9)	1288 (1020–1662)	0.001	1948.2 (1509.1–2438.3)	1567.1 (1097.0–2049.6)	0.021
LPC(18:0)	63,379.6 (44,850.8–85,600.2)	47,396.8 (37,198.6–62,924.2)	0.020	56,000.6 (40,633.9–80,246.4)	49,921.7 (37,941.0–72,519.6)	0.308
LPC(18:1)	21,454.3 (15,067.6–27,481.6)	21,789 (19,151–29,051)	0.233	20,582.1 (15,193.3–26,645.2)	26,269.2 (19,120.5–32,008.3)	0.034
LPC(18:2)	27,509.3 (23,480.1–35,012.6)	43,606 (36,613–51,253)	0.000	29,714.0 (24,478.3–35,012.0)	44,172.4 (37,096.7–54,805.1)	0.000
LPC(18:3)	13,616.8 (11,638.8–15,873.7)	11,489 (9480–12,909)	0.000	13,153.1 (11,447.2–14,964.1)	11,987.2 (9410.4–14,232.1)	0.019
LPC(20:0)	240.3 (195.0–2721.8	179.9 (128.8–214.5)	0.003	211.5 (142.3–300.6)	195.2 (147.0–250.7)	0.209
LPC(20:2)	431.6 (223.3–603.5)	505.6 (324.2–729.5)	0.140	332.2 (273.4–565.4)	538.1 (413.1–763.0)	0.005
LPC(20:3)	9995.9 ± 3311.8	10,234.2 ± 2985.6	0.733	10,128.2 ± 3877.3	11,989.3 ± 4074.7^#^	0.031
LPC(20:4)	10,415.2 (9050.0–12,533.2)	22,367.1 (18,945.2–27,351.0)	0.000	9537.0 (8019.2–13,176.2)	23,141.1 (18,657.2–30,853.1)	0.000
LPC(20:5)	5946.8 (4188.1–6098.2)	7162.1 (6231.0–8515.7)	0.000	5548.6 (4449.0–6600.0)	7380.1 (6296.8–8854.0)	0.000
LPC(22:6)	2154.4 (1496.9–2841.6)	4731 (3964–7186)	0.000	2039.1 (1394.3–2047.0)	5763.1 (4396.7–7838.1)	0.000
LPE(16:0)	575.5 (437.8–733.7)	506.9 (409.4–689.7)	0.688	685.2 (530.7–882.2)*	646.4 (447.8–1000.5)	0.311
LPE(18:0)	632.9 (434.0–876.8)	322.9 (229.4–417.5)	0.000	682.3 (462.8–955.6)	362.4 (255.5–475.9)	0.000
LPE(O-20:0)	326.1 ± 150.4	232.3 ± 110.2	0.002	324.5 ± 196.0	283.1 ± 187.0	0.343
LPI(18:0)	286.1 (145.7–426.6)	649.0 (545.0–1019.9)	0.000	266.2 (166.9–538.2)	770.2 (591.7–1063.6)	0.000
LPI(20:4)	135.1 (8.31–214.0)	642.8 (505.1–772.2)	0.000	156.7 (117.3–238.0)	750.9 (552.6–883.7)	0.000
LPS(O-18:0)	161.9 (102.2–189.9)	137.1 (111.0–179.0)	0.236	156.6 (79.3–230.5)	84.9 (62.7–126.7)	0.010
PC(O-36:3)	2406.3 (1419.2–4111.8)	1645.2 (1028.1–2062.1)	0.010	2601.0 (2043.8–3684.2)	1416.1 (978.2–2124.0)	0.000
PC(34:0)	3277.6 (2676.6–3817.9)	2862.6 (2007.3–3850.8)	0.525	2615.7 (1906.1–3682.2)*	2662.1 (2300.6–3108.3)	0.128
PC(36:4)	93,069.1 (79,780.3–136,743.0)	102,356.0 (74,512.1–151,229.2)	0.677	85,712.1 (69,863.2–123,458.7)	82,948.5 (64,544.1–106,702.8)	0.881
PC(38:4)	62,078.4 (51,420.4–81,452.7)	60,376.6 (49,836.6–88,052.3)	0.544	51,559.8 (45,042.3–69,994.4)*	54,934.6 (43,751.4–67,162.3)	0.693
PC(P-38:5)	1997.1 (1525.7–3132.2)	1076.7 (545.6–1717.7)	0.000	1868.1 (1443.7–2673.1)	834.8 (584.1–1232.5)	0.000
PC(40:5)	4708.1 (2919.5–6161.2)	4472.5 (2727.7–7600.9)	0.694	3020.5 (1615.0–5141.7)*	3764.6 (2770.9–4453.6)	0.267
PC(40:8)	5548.6 (3987.5–8139.5)	4550.1 (2762.6–6087.2)	0.048	5139.6 (3316.1–6483.0)	4002.0 (3025.1–5229.7)	0.213
PC(40:9)	1262.7 (704.9–1563.1)	1222.2 (831.9–1621.9)	0.861	1022.2 (766.7–1213.7)	770.0 (549.5–1103.0)	0.073
PI(34:2)	5696.3 (3559.2–11,738.2)	3535 (2142–6030)	0.008	7266.5 (3857.6–14,957.1)	4547.6 (1833.0–10,994.1)	0.033
PI(36:2)	5850.2 (4289.3–8214.6)	5376.2 (3834.7–7459.8)	0.328	6546.3 (4829.2–8008.2)	4412.7 (3257.6–6519.1)	0.010
PI(36:3)	1196.8 (774.1–1642.2)	929.8 (732.7–1315.8)	0.212	1156.7 (863.7–1461.1)	867.2 (592.8–1153.4)	0.034
PI(36:4)	13,953.2 (4650.9–25,982.1)	5958.3 (3447.4–15,387.2)	0.040	13,822.2 (5681.0–29,772.2)	7950.0 (3106.8–24,503.1)	0.225
PI(38:4)	26,434.9 (18,158.4–38,048.0)	23,546.5 (16,653.2–33,951.2)	0.305	29,146.6 (22,843.2–36,429.2)	21,230.1 (15,293.6–31,947.8)	0.018
PI(38:6)	2417.7 (1479.4–48,710.8)	1712.3 (1181.8–3517.3)	0.073	3413.8 (2439.5–5993.4)	2308.4 (1689.1–3363.4)	0.014
PI(40:6)	794.9 (589.5–1326.9)	543.3 (421.2–829.7)	0.003	932.1 (680.0–1457.6)	646.1 (489.2–1081.1)	0.016
PS(P-36:1)	4455.3 (2066.7–7302.3)	2056.2 (1040.2–3618.7)	0.003	3341.2 (2557.0–5854.9)	2351.0 (1410.1–3852.2)	0.021
Triglyceride (pmol/ml)						
TG(46:11)	247.4 (132.5–668.7)	121.2 (87.3–418.6)	0.216	219.2 (156.8–517.2)	194.1 (89.2–284.2)	0.005
TG(48:0)	290.9 (117.6–594.5)	250.8 (83.1–535.7)	0.068	482.4 (135.7–1054.5)	463.8 (103.9–1081.2)^#^	0.199
TG(P-52:1)	26.5 (18.8–56.7)	27.7 (14.7–40.9)	0.919	42.0 (25.7–66.0)	42.8 (22.5–65.5)^#^	0.618
TG(52:7)	275.6 (148.7–418.7)	215.2 (128.7–335.2)	0.007	320.1 (154.0–762.8)	311.2 (172.1–518.0)^#^	0.116
TG(54:8)	711.3 (419.8–1176.3)	579.2 (284.1–909.1)	0.053	937.5 (433.3–2118.2)	913.9 (433.4–1363.6)^#^	0.074

The data were presented as the mean ± SD, except for skewed variables, which were presented as the median with the interquartile range given in parentheses. The unit of lipids in this table was pmol/mL, except for free fatty acids which were 1

* P < 0.05 when comparing berberine plus lifestyle intervention group and lifestyle intervention alone group at baseline

^#^ *P* < 0.05 when comparing berberine plus lifestyle intervention group and lifestyle intervention alone group after 16-week treatment

More lipids were significantly changed after berberine treatment compared with lifestyle intervention. Nineteen free fatty acids [FA(15:1), FA(16:1), FA(16:2), FA(16:3), FA(18:1), FA(18:2), FA(20:2), FA(20:3), FA(20:4), FA(20:5), FA(22:4), FA(22:5), FA(22:6), FA3, FA40, FA48, FA51, FA(α-18:3), FA(γ-18:3)] were markedly decreased in the BBR group, and 8 of these free fatty acids were also altered after lifestyle intervention. Levels of Cer(d18:1/18:0), Cer(d18:1/20:0) and Cer(d18:1/18:0)-1P sphingolipids were significantly decreased. The sphingomyelin (SM) SM(d18:1/16:1) was significantly decreased, and SM(d18:1/24:4) was elevated. Levels of LPE(18:0), LPE(O-20:0), PC(O-36:3), PC(P-38:5), PC(40:8), PS(P-36:1), 5 lysophosphatidylcholine [LPC(14:0), LPC(16:1), LPC(18:0), LPC(18:3), LPC(20:0)], 3 phosphatidylinositol [PI(34:2), PI(40:6), PS(P-36:1)] and TG(52:7) were markedly decreased, and LPI(18:0), LPI(20:4), PC(P-38:5) and 4 lysophosphatidylcholines [LPC(18:2), LPC(20:2), LPC(20:4), LPC(20:5), LPC(22:6)] were significantly elevated (Table 2).

Table 2 shows that most lipids were markedly decreased after LSI. Levels of 8 free fatty acids [FA(16:1), FA(16:2), FA(18:2), FA(20:2), FA(20:3), FA27, FA51, FA(α-18:3)] were significantly decreased. The levels of LPC(16:1), LPC(18:3), LPE(18:0), LPS(O-18:0), PC(O-36:3), PC(P-38:5) and 6 phosphatidylinositols [PI(34:2), PI(36:2), PI(36:3), PI(38:4), PI(38:6), PI(40:6)], PS(P-36:1) were significantly decreased, and LPI(18:0), LPI(20:4) and 7 lysophosphatidylcholine [LPC(18:1), LPC(18:2), LPC(20:2), LPC(20:3), LPC(20:4), LPC(20:5), LPC(22:6)] levels were markedly elevated. The TG (46:11) level was also significantly decreased.

Only 5 lipids were significantly different before BBR treatment or LSI: LPE (16:0), PC(34:0), PC(38:4), PC(40:5) and SM(d18:1/12:0). However, 10 lipids were markedly different after the two interventions. Levels of Cer(d18:1/28:0)-1-P, LPC(14:0), LPC(20:3) and 4 triglycerides [TG(48:0), TG(P-52:1), TG(52:7), TG(54:8)] in the berberine treatment group were significantly lower than the lifestyle intervention group. Levels of SM(d18:1/24:4), PC(36:4) and PC(40:9) in the berberine treatment group were significantly higher than the lifestyle intervention group.

These results demonstrated that the lipid-lowering effect of berberine was similar with lifestyle intervention. Both treatments regulated various types of lipids in metabolic pathways. The two interventions similarly regulated free fatty acids, phosphoglycerides and glycerides, but there were obvious differences in regulation for sphingolipids. Ceramide and ceramide-1-phosphate levels decreased markedly after BBR treatment, and sphingomyelin levels were slightly elevated. The lifestyle intervention only significantly decreased sphingosine levels. These data suggest that berberine participates in phospholipid metabolism.

**Fig. 2:**
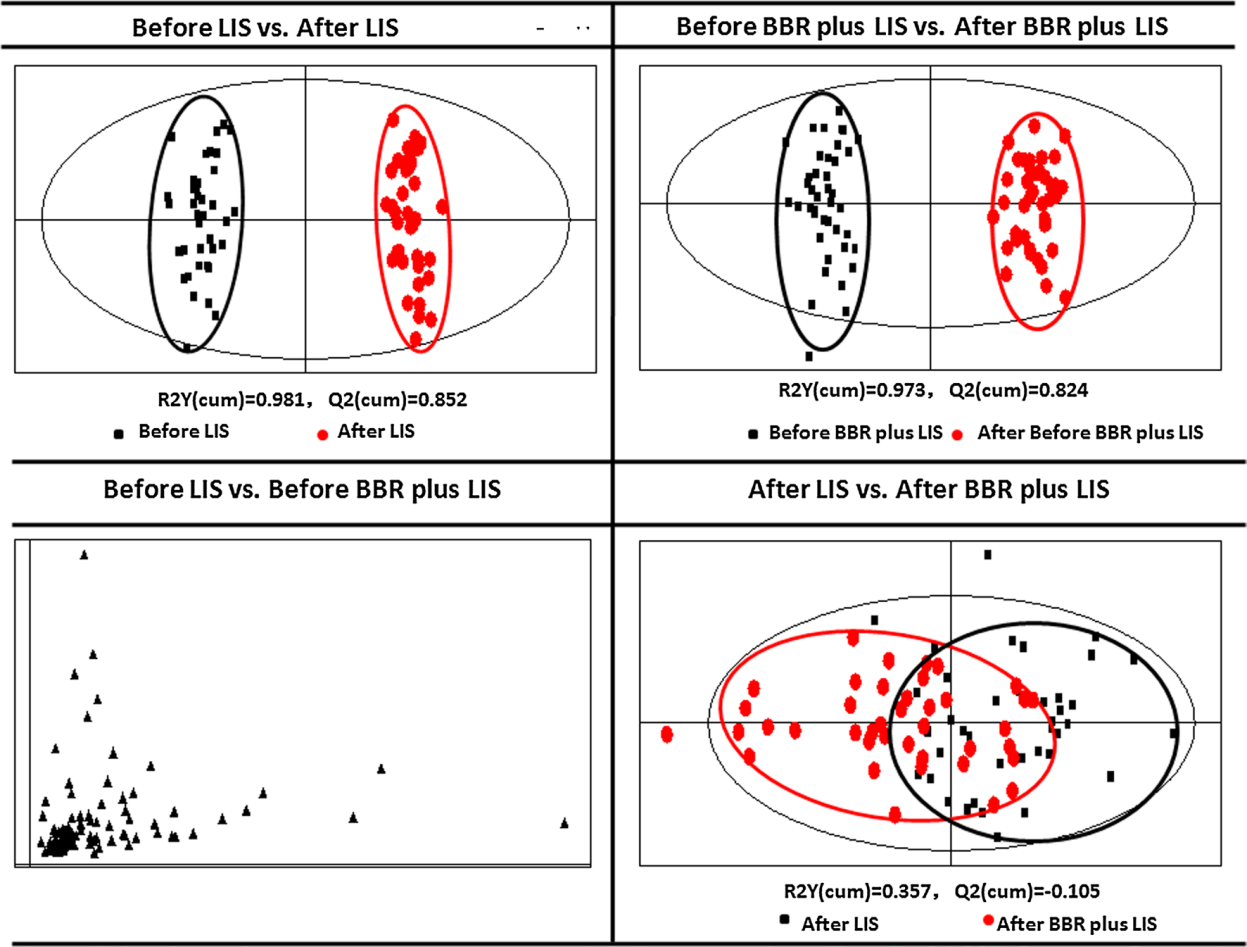
In order to further study the subtle differences between groups, orthogonal partial least squares discriminant analysis (OPLS-DA) was employed. This figure was the* score plots* obtained from OPLS-DA

Orthogonal partial least squares discriminant analysis (OPLS-DA) was used to further study the subtle differences between groups. Figure 2 shows the score plots obtained from OPLS-DA. Patient groups before and after lifestyle intervention exhibited a reliable discrimination, which means lifestyle intervention obviously affected lipid metabolic pathways. OPLS-DA analysis detected four biomarkers that reflected the therapeutic effect of lifestyle intervention (Table 3).

**Table 3:** Lipid biomarkers found in patients’ plasma

Before LIS vs. after LIS	Before BBR plus LIS vs. after BBR plus LIS	After LIS vs. after BBR plus LIS
LPC(20:4)	LPC(18:2)	PC(36:4)
LPC(22:6)	LPC(20:4)	PC(40:9)
LPI(20:4)	LPC(20:5)	
PC(P-38:5)	LPC(22:6)	
	LPI(20:4)	
	LPI(18:0)	

Based on the criteria for the identification of the potential biomarkers, OPLS-DA analysis detected some biomarkers reflecting the therapeutic effect of berberine and lifestyle intervention

The berberine treatment group also exhibited an obvious discrimination, which means that berberine markedly regulated the lipid metabolic pathways. Six biomarkers that reflected the therapeutic effect of berberine were detected (Table 3).

The two groups of patients could not be discriminated before intervention. The two groups only achieved incomplete discrimination after intervention. Only two biomarkers were detected that discriminated these two types of intervention (Table 3).

## Discussion

In the present study, BBR treatment for 16 weeks reduced more hepatic fat content in NAFLD patients, which was paralleled with a global metabolic benefit, as reflected in reduced body weight and improved glucose and lipid profiles compared with lifestyle intervention alone. Lipidomics analyses demonstrated that berberine and lifestyle intervention alone regulated various types of lipids in lipid metabolic pathways comprehensively. Notably, berberine exerted a special effect on sphingolipids, including a down-regulation of ceramides, which play an important role in the pathogenesis of nonalcoholic fatty liver disease. However, lifestyle intervention alone had no effect on ceramides.

Patients treated with berberine lost significantly more liver fat content and exhibited greater reductions in blood glucose, triglycerides and cholesterol than the LSI group, which is consistent with previous studies [1, 2, 7]. BBR was absorbable after oral administration in our previous study [9], which demonstrated that BBR directly affected hepatic lipid metabolism.

Previous studies indicated that fat deposit in hepatocytes was associated with several changes in circulating lipidomes [11]. Lipids are the fundamental components of cellular membranes, and they are essential because they represent the biochemical activity signature during lipid metabolism. Therefore, lipids are closely related to observable phenotypes. Lipidomics is the process of defining multivariate lipid metabolic trajectories that represent the systemic response (i.e., holistic lipid metabolic changes) of a living system to pharmaceutical interventions over time.

Berberine plus lifestyle intervention and lifestyle interventional one exhibited a substantially greater effect on serum lipid metabolism, which primarily included FA, LPC, LPI, LPE, PC and PI (Table 2). Only berberine plus lifestyle intervention altered serum sphingolipids, including decreasing serum sph(d18:1), Cer(d18:1/18:0), Cer(d18:1/18:0)-1-p, and Cer(d18:1/20:0) levels, which was not achieved by exercise with diet control alone. This effect may be a special mechanism of berberine.

Ceramides are important members of the sphingolipid family, and they are essential precursors for complex sphingolipids. Ceramide and ceramide-derived sphingolipids are structural components of membranes, and these components are associated with insulin resistance, oxidative stress, and inflammation [17–19], which suggest that they play a role in the development of liver steatosis [20, 21]. Ceramides (Cer) may inhibit several mediators of the insulin signaling pathway, including insulin receptor substrate 1 (IRS1), phosphatidylinositol 3-kinase (PI-3 K) and AKt/PKB [22]. Previous studies indicated that inhibition of ceramide synthesis, including ceramide-1-phosphate and glucosylceramide, inhibited several underlying causes of insulin resistance and improved insulin sensitivity in tissues [23–26]. A recent report suggested that the liver is a major contributor of circulating ceramide species [21]. Plasma levels of total ceramide and all ceramide species in western-diet induced NAFLD were elevated, and major changes were observed in Cer(d18:1/16:0), Cer(d18:1/22:0), Cer(d18:1/24:0) and Cer(d18:1/24:1). A study in animals demonstrated that inhibition of ceramide biosynthesis reduced hepatic and plasma ceramides and sphingomyelin, improved insulin sensitivity and reduced hepatic fat accumulation [27, 28]. Berberine significantly decreased the level of ceramides in the present study, with major changes in Cer(d18:1/18:0), Cer(d18:1/20:0) and Cer(d18:1/18:0)-1P, which demonstrates that the improving effects of BBR in liver steatosis related to ceramide reduction.

Berberine also reduced blood glucose levels in the oral glucose tolerance test (OGTT), which is consistent with previous studies [7]. Some studies suggested that berberine decreased blood glucose by improving insulin resistance [7]. A recent report suggested that overexpression of acid ceramidase in the liver reduced hepatic ceramide levels and improved hepatic and adipose insulin sensitivity [21]. Plasma ceramide levels reflected changes in hepatic ceramide levels. The present study found that berberine reduced circulating ceramide levels, which may be related to decreasing serum glucose.

Lipid microdomains or caveolae, which are small invaginations of plasma membrane, emerged as important elements for lipid uptake, including triglycerides (TG) and fatty acids [29]. Sphingomyelin (SM) is a major phospholipid of lipid microdomains. The conversion of SM to Cer is also necessary to maintain the homeostasis of these domains [30]. Sphingomyelin synthase (SMS) converts Cer to SM in the plasma membrane, and a deficiency of SMS may affect the metabolism of ceramide, sphingosine and sphingosine 1-phosphate. The present study found that berberine also altered plasma levels of SM, as characterized by a significant decrease in SM(d18:1/16:1) and elevated SM(d18:1/12:0) and SM(d18:1/24:4).BBR also reduced the level of ceramides. However, how these changes affect hepatic fat deposit requires investigation.

PC combats fatty liver and blood lipid disorders due to obesity [31, 32], promotes the absorption and utilization of lipid, removes cholesterol from vessel walls, reduces HDL-cholesterol and promotes hydrolysis of atherosclerotic plaques [33]. The content ratio of PC and PE is closely related to the accumulation of TG in liver [34]. Berberine treatment and lifestyle intervention regulated the level of PC in plasma. Two PCs containing polyunsaturated fatty acids [PC(36:4) and PC(40:9)] were biomarkers that discriminated between these two interventions, and the levels of these PCs in the berberine group were significantly higher than the lifestyle intervention.

Lysophosphatidylcholine (LPC) has been reported to be closely related to many inflammatory diseases, such as T2DM, obesity, and atherosclerosis [35]. Several LPC biomarkers [LPC(18:2), LPC(20:4), LPC(20:5), and LPC(22:6)] were significantly elevated after BBR treatment, and other LPCs [LPC(14:0), LPC(16:1), LPC(18:0), LPC(18:3), and LPC(20:0)] were reduced. Lifestyle intervention also caused similar changes. The levels of 2 lysophosphatidylinositol (LPI) biomarkers [LPI(18:0) and LPI(20:4)] also markedly increased after berberine and lifestyle intervention alone treatments. The role of this lipid type has not been reported in type 2 diabetes or nonalcoholic fatty liver disease.

Further studies are needed to elucidate the biological mechanisms accounting for the link between berberine, serum lipid profile, especially ceramides, and fatty liver, but these findings indicate that the systematic analysis of serum lipid species, rather than lipid classes as a whole, may reveal the beneficial effects of berberine on fatty liver beyond improvements in clinical biomarkers.

One limitation of the present study is the cross-sectional nature of the study, and no patients were examined using liver biopsy because of ethical concerns. Therefore, the effects of BBR on human hepatic lipid profile and the genes related to lipid metabolism require further study.

## Conclusions

The application of LC-MS-based lipidomics and measurement of biochemical parameters revealed the differential therapeutic effects of berberine and mere lifestyle intervention on serum lipid profile, which were comparable with their differential effects on hepatic fat content, serum lipid and glucose metabolism. Berberine more substantially altered serum lipid species compared with mere lifestyle intervention. The altering of sphingolipid metabolism by BBR, including a decrease in serum ceramides, was a novel mechanism. These findings suggest that a lipidomics approaches useful for the elucidation of the complex mechanism of action of particular drugs and a novel tool to probe the mechanisms of NAFLD progression. Future studies are required to precisely evaluate the predictive findings in additional cohorts and confirm whether we identified an early marker of NAFLD and its associated therapeutics.

## Additional files

10.1186/s12967-016-0982-x Changes of clinical and biochemical parameters after treatment.
10.1186/s12967-016-0982-x The line graph of the glucose tolerance test (0–3 h). Data were mean ± SD, LSI: lifestyle intervention, BBR plus LSI: berberine treatment plus lifestyle intervention. **P* < 0.05 when comparing before and after berberine plus lifestyle intervention treatment, #*P* < 0.05 when comparing before and after lifestyle intervention alone treatment.

## Abbreviations

BBR: berberine
TC: total cholesterol
TG: triglyceride
LDL-c: low-density lipoprotein
NAFLD: nonalcoholic fatty liver disease
DG: diacylglycerol
PC: phosphatidylcholine
PUFAs: polyunsaturated fatty acids
NASH: nonalcoholic steatohepatitis
IGR: impaired glucose regulation
LSI: lifestyle intervation
HFC: hepatic fat content
BMI: body mass index
FA: fatty acid
HexCer: glycoceramide
LPA: lyso-phosphatidic acid
LPC: lyso-phosphatidylcholine
LPE: lyso-phosphatidylinositol
LPI: lyso-phosphatidylinositol
PA: phosphatidic acid
PC: phosphatidylcholine
PE: phosphatidylethanolamine
PI: phosphatidylinositol
PS: phosphatidylserine
SM: sphingomyelin
Sph: sphingosine
Cer: ceramide
Cer-1P: phosphoceramide
dhCer: dihydroceramide
dhCer-1P: phosphodihydroceramide
dhSph: dihydrosphingosine
ER: endoplasmic reticulum
