# Supplementary material for: Lipid profiling of the therapeutic effects of berberine in patients with nonalcoholic fatty liver disease
Source: J Transl Med. 2016 Sep 15;14:266. doi: 10.1186/s12967-016-0982-x (PMC5024486; doi:10.1186/s12967-016-0982-x)
Supplement: Supplementary file 1 — 10.1186/s12967-016-0982-x Changes of clinical and biochemical parameters after treatment. [file 12967_2016_982_MOESM1_ESM.docx]

**Table S1. Changes of clinical and biochemical parameters after treatment**

|  | | **BBR plus LSI**  **(n=41)** | | **LSI**  **(n=39)** | **P value#** |
| --- | --- | --- | --- | --- | --- |
|  |  |  |  |  | **BBR plus LSI vs. LSI** |
| Weight (kg) | -4.0(-6.0~-1.3)* | | -2.0(-3.7~-0.5)* | 0.013 |  |
| BMI(kg/m^2^) | | -1.4(-2.3~0.5)* | | -0.7(-1.3~-0.2)* | 0.007 |
| Waist (cm) | | -4.3(-7.5~-1.6)* | | -2.0(-4.0~0.0)* | 0.043 |
| HFC(%) | | -15.4（-27.1 ~4.5）* | | -11.3(-18.4~-1.4)* | 0.021 |
| Blood glucose (mmol/L) | | | | |  |
| 0min | | -0.3(-1.0 ~0.1) * | | -0.1(-0.4~0.3) | 0.324 |
| 30min | | -1.4(-2.4 ~1.0) * | | -0.2(-1.8~0.6) | 0.433 |
| 60min | | -1.7(-3.1 ~-0.2) * | | -1.2(-2.7~0.7) * | 0.368 |
| 120min | | -1.8(-4.3 ~-0.2) * | | -0.4(-2.3~1.2) | 0.092 |
| 180min | | -1.7(-3.8 ~-0.1) * | | -0.4(-1.4~0.7)* | 0.013 |
| AUCg | | -5.9(-6.9~-4.8)* | | -4.0(-4.6~-1.9)* | 0.041 |
| HbA1c(%) | | -0.3(-1.0~-0.2)* | | -0.1(-0.4~0.0) | 0.302 |
| Serum insulin (mU/mL) | | | |  |  |
| 0min | | -1.1(-5.0~2.8) | | -1.7(-4.6~1.3) | 0.900 |
| 30min | | 3.0(-7.6~21.1) | | 0.8(-23.6~18.3) | 0.911 |
| 120min | | 0.3(-23.6~20.1) | | -6.7(-32.1~32.5) | 0.541 |
| HOMA-IR | | 0.5(-1.0~1.8) | | 0.2(-0.5~1.4) | 0.552 |
| HOMAβ | | 0.3(-23.6~20.1) | | 3.7(-14.7~17.2) | 0.645 |
| ΔI30/ΔG30 | | -2.4(-8.8~0.4)* | | -1.0(-4.8~5.4) | 0.126 |
| TC(mmol/L) | | | -0.5(-1.0~-0.1)* | 0.0(-0.4~0.4) | 0.002 |
| TG(mmol/L) | | | -0.5(-1.0~0.0)* | 0.0(-0.5~0.3) | 0.013 |
| HDL-c(mmol/L) | | | 0.0(-0.1~0.1) | 0.0(-0.1~0.1) | 0.432 |
| LDL-c(mmol/L) | | | -0.3(-0.8~0.4)* | 0.0(-0.4~0.3) | 0.317 |
| APO-A(g/L) | | | -0.1(-0.3~0.1)* | 0.0(-1.7~0.3) | 0.028 |
| APO-B(g/L) | | | -0.1(-0.2~0.0)* | -0.0(-0.1~0.1) | 0.321 |
| APO-E(mg/L) | | | -5.5(-17.0~-1.8) | 0.0(-4.0~4.8) | 0.428 |
| LP(a)(mg/L) | | | 12.5(-21.5~57.3)* | 10.0(-29.5~28.0) | 0.349 |
| Liver enzyme (U/L) | | | | |  |
| ALT | | -14.0(-21.5~-5.5)* | | -11.0(-20.5~-0.8)* | 0.073 |
| AST | | -5.0(-10.0~-1.0)* | | -4.0(-10.0~0.5)* | 0.906 |
| γ-GT | | -6.0(-26.0~-1.5)* | | -4.0(-17.3~2.5)* | 0.217 |

Data were presented as the median with the interquartile range given in parentheses. LSI: lifestyle intervention, BBR plus LSI: berberine treatment plus lifestyle intervention. * *P*<0.05 when comparing before and after treatment, # *P* value after adjustment for age, BMI, baseline data.
