# Supplementary figures and images for: Lipid profiling of the therapeutic effects of berberine in patients with nonalcoholic fatty liver disease
Source: J Transl Med. 2016 Sep 15;14:266. doi: 10.1186/s12967-016-0982-x (PMC5024486; doi:10.1186/s12967-016-0982-x)

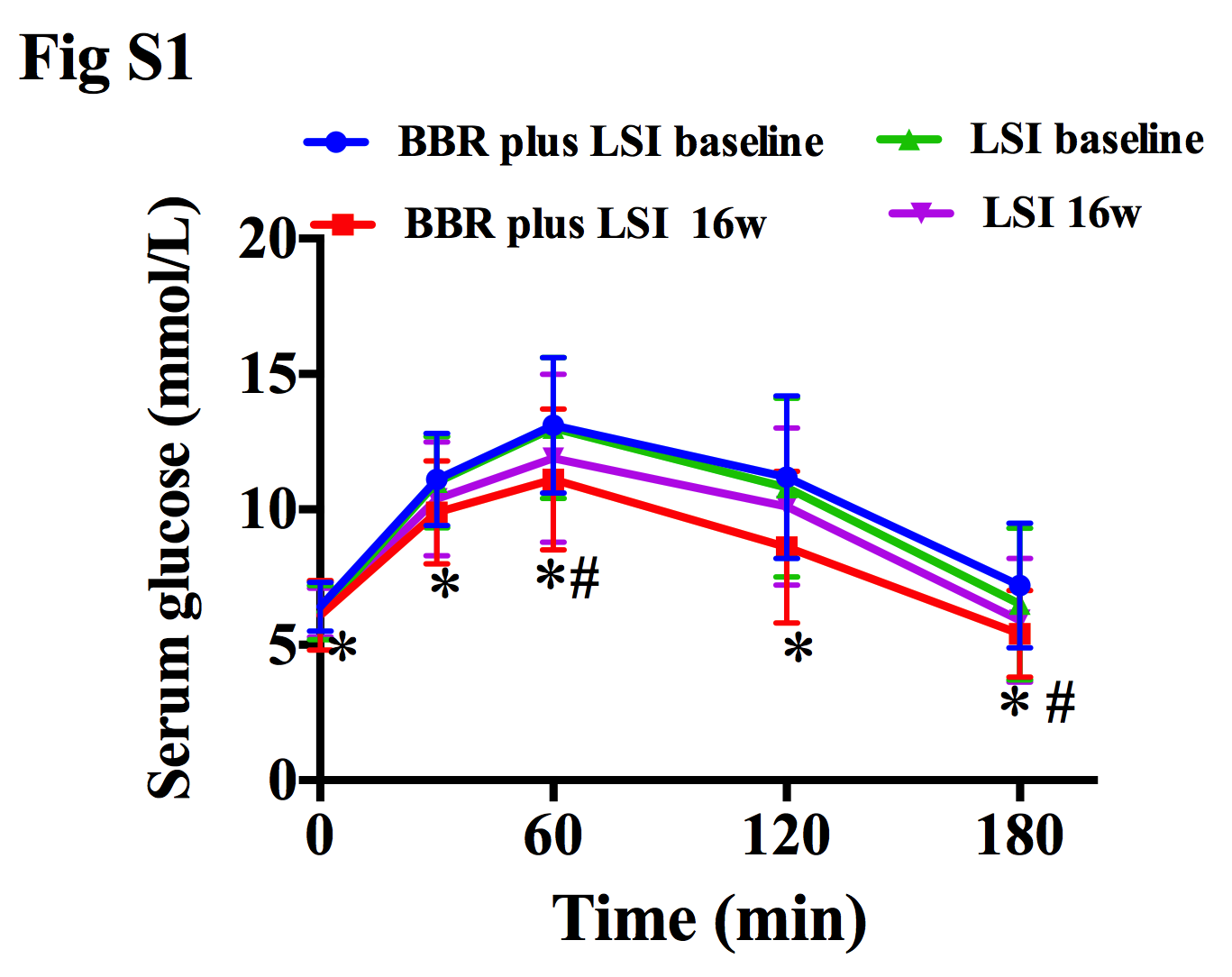

Supplement: Supplementary file 2 — 10.1186/s12967-016-0982-x The line graph of the glucose tolerance test (0–3 h). Data were mean ± SD, LSI: lifestyle intervention, BBR plus LSI: berberine treatment plus lifestyle intervention. *P < 0.05 when comparing before and after berberine plus lifestyle intervention treatment, #P < 0.05 when comparing before and after lifestyle intervention alone treatment. [file 12967_2016_982_MOESM2_ESM.tiff]
